# Population genetic structure of the predatory, social wasp *Vespula pensylvanica* in its native and invasive range

**DOI:** 10.1002/ece3.1757

**Published:** 2015-11-13

**Authors:** Linh M. Chau, Cause Hanna, Laurel T. Jenkins, Rachel E. Kutner, Elizabeth A. Burns, Claire Kremen, Michael A. D. Goodisman

**Affiliations:** ^1^ School of BiologyGeorgia Institute of TechnologyAtlantaGeorgia30332; ^2^ Environmental Science and Resource ManagementCalifornia State UniversityCamarilloCalifornia93012; ^3^ Environmental Science, Policy and ManagementUniversity of CaliforniaBerkeleyCalifornia94720

**Keywords:** Archipelago, biological invasion, bottleneck, genetic diversity, microsatellites, *Vespula*

## Abstract

Invasive species cause extensive damage to their introduced ranges. Ocean archipelagos are particularly vulnerable to invasive taxa. In this study, we used polymorphic microsatellite markers to investigate the genetic structure of the social wasp *Vespula pensylvanica* in its native range of North America and its introduced range in the archipelago of Hawaii. Our goal was to gain a better understanding of the invasion dynamics of social species and the processes affecting biological invasions. We found that *V. pensylvanica* showed no significant genetic isolation by distance and little genetic structure over a span of 2000 km in its native range. This result suggests that *V. pensylvanica* can successfully disperse across large distances either through natural‐ or human‐mediated mechanisms. In contrast to the genetic patterns observed in the native range, we found substantial genetic structure in the invasive *V. pensylvanica* range in Hawaii. The strong patterns of genetic differentiation within and between the Hawaiian Islands may reflect the effects of geographic barriers and invasion history on gene flow. We also found some evidence for gene flow between the different islands of Hawaii which was likely mediated through human activity. Overall, this study provides insight on how geographic barriers, invasion history, and human activity can shape population genetic structure of invasive species.

## Introduction

Invasive species are recognized as one of the top threats to the environment (Sakai et al. 2001; Pejchar and Mooney 2009; Kirk et al. 2013; Simberloff et al. 2013). Introduced species can displace native taxa, alter habitats, act as vectors for foreign diseases, and reduce levels of biodiversity (Sakai et al. 2001; Kenis et al. 2009; Brockerhoff et al. 2010; Beggs et al. 2011). Invasive species are often transported to new locations through human‐mediated methods (Sakai et al. 2001). Consequently, the growing rate of globalization has increased the risk of non‐native species being introduced to new regions (Pejchar and Mooney 2009).

Many social insects are highly successful invasive species (Moller 1996; Tsutsui et al. 2000; Chapman and Bourke 2001; Tsutsui and Suarez 2003; Beggs et al. 2011; Husseneder et al. 2012; Evans et al. 2013; Kirk et al. 2013; Gotzek et al. 2015). Introductions of invasive termites, ants, social bees, and social wasps have caused substantial damage to local ecosystems and economies (Holway et al. 2002; Suarez and Case 2002; Beggs et al. 2011; Evans et al. 2013). The success of social insects as invasive species is likely associated with their social structure, in addition to other factors such as their occupation of broad niches, high dispersal power, and effective predator defense (Moller 1996). These traits allow invasive social insects to work efficiently and rapidly increase in density in new environments, raising the likelihood of invasion success (Moller 1996; Smith et al. 2008).

*Vespula* wasps are particularly notorious invasive social insects. *Vespula* wasps are native to various regions throughout the northern hemisphere but have been introduced to many locations, such as Australia, South America, Hawaii, and New Zealand (Akre et al. 1981; Brockerhoff et al. 2010; Beggs et al. 2011; Monceau et al. 2014). Introductions of *Vespula* wasps have led to negative consequences for their invasive ecosystems (Matthews et al. 2000; Beggs et al. 2011). For example, *Vespula* species are known to compete with native pollinators and carnivores (Brockerhoff et al. 2010; Elliott et al. 2010; Beggs et al. 2011; Hanna et al. 2014b). This phenomenon has serious costs and has been linked to the decline of native taxa (Elliott et al. 2010).

The western yellowjacket, *Vespula pensylvanica*, has emerged as one of the most destructive invasive *Vespula* species. *V. pensylvanica* is native to the western parts of North America but was recently introduced to all of the major islands of Hawaii (Nakahara 1980; Akre et al. 1981; Visscher and Vetter 2003). The introduction of *V. pensylvanica* to Hawaii has had serious consequences for native Hawaiian fauna. As Hawaii has no native social insects, introduced *V. pensylvanica* have no direct, native, social insect competitors (Wilson 1996). Thus, the introduction of *V. pensylvanica* into Hawaii has led to the displacement of endemic insects and pollinators, such as the Hawaii picture wing fly and *Hylaeus* bees (Foote and Carson 1995; Wilson and Holway 2010; Hanna et al. 2014b). The ecological effects of *V. pensylvanica* are possibly magnified by the increased population density that stems from perennial nests that are common in Hawaiian populations (Nakahara 1980; Gambino 1991; Hanna et al. 2014a).

The purpose of this study was to use genetic markers to gain a greater understanding of the invasion of Hawaii by *V. pensylvanica*. The historical records of the invasion and presumed consequences of species invasions allow us to make predictions about the population genetic structure of invasive and native *V. pensylvanica*. For example, we expect that invasive populations will harbor less variation than native populations, as is typical for introduced species (Dlugosch and Parker 2008). In addition, we expect that some introduced populations may show evidence of population bottlenecks, which occur if populations undergo reductions in size during the founding process (Cornuet and Luikart 1996; Luikart et al. 1998).

We also predict that *V. pensylvanica* will display genetic isolation by distance across its native range, given the broad distribution of *V. pensylvanica* across western North America and the presumed limited dispersal ability of *Vespula* queens (Masciocchi and Corley 2013). In contrast, we expect little genetic isolation by distance within islands in Hawaii. Introduced Hawaiian populations are believed to have been recently founded from multiple, distinct introduction events, which would be expected to obscure patterns of genetic isolation by distance (Nakahara 1980).

Finally, we predict differences in genetic relationships between *V. pensylvanica* populations on the western Hawaiian Islands of Kauai, Oahu, and the eastern Hawaiian Islands of Molokai, Lanai, Hawaii, and Maui. Populations on Molokai, Lanai, Hawaii, and Maui were colonized in the late 1970s (Nakahara 1980). These populations were thought to have been established by Christmas trees shipments from Oregon (Nakahara 1980). So we expect that these eastern populations will be closely related to each other. In contrast, *V. pensylvanica* was first noted on Kauai and Oahu in 1919 and 1936, respectively (Nakahara 1980). Thus, we predict that the populations on Kauai and Oahu will be less related to each other, and to the populations on the eastern islands.

Overall, the goal of this study was to understand the invasion of *V. pensylvanica* across the Hawaiian Islands. Archipelagoes, like Hawaii, serve as models for investigating the interplay between ecological and evolutionary processes in shaping invasion dynamics because they vary in shape, size, degree of isolation, and age (Drake et al. 2002). We are interested in determining how geographic barriers affect population structure and genetic variation in native and invasive habitats. Ultimately, we hope this study will provide insight into the role of geography and the effects of humans on biological invasions.

## Methods

### Sampling scheme

**Figure 1:**
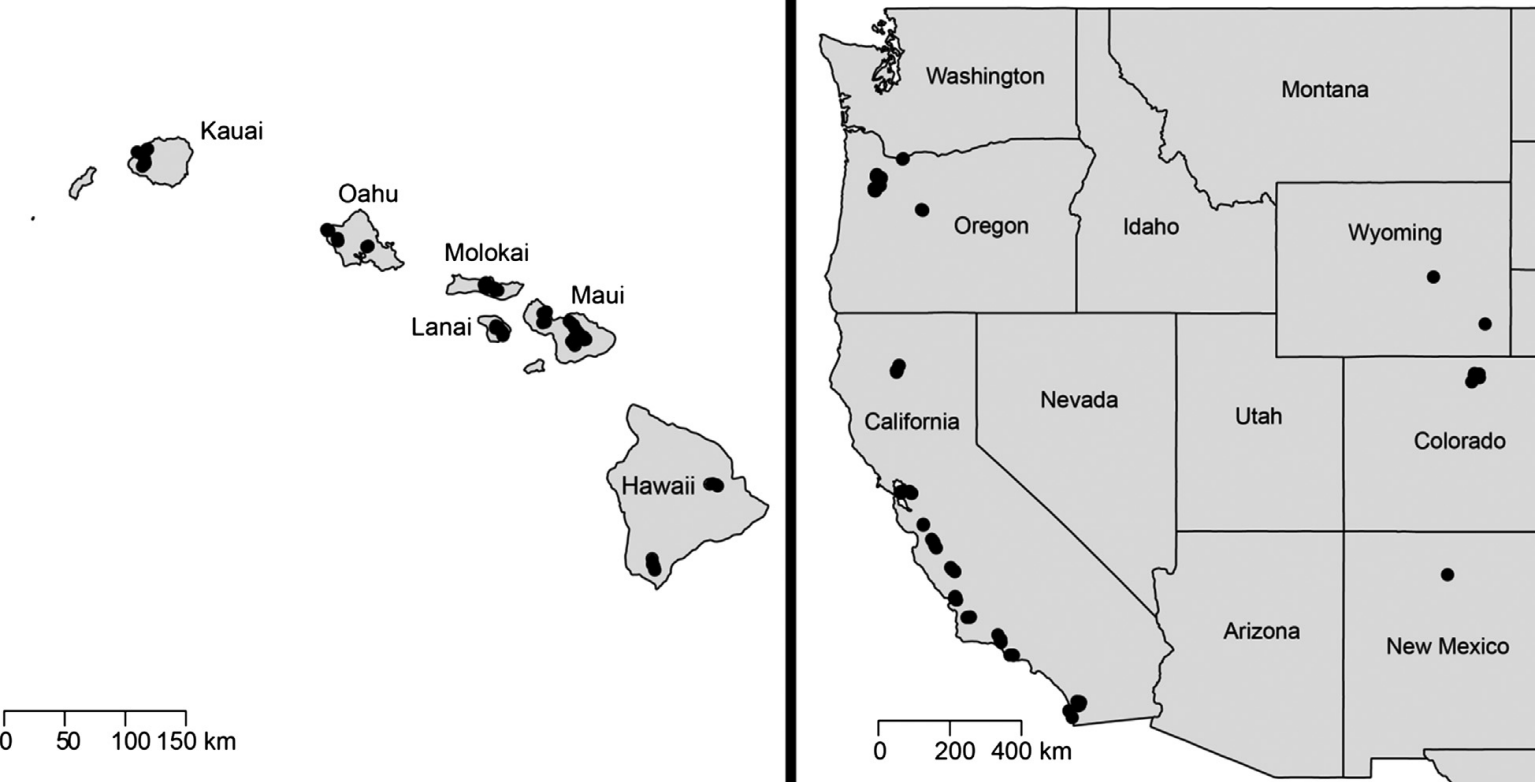
Locations of *Vespula pensylvanica* traps in the invasive (Hawaiian) and native (mainland) range.

We collected 1364* V. pensylvanica* workers from their native range in the western part of the United States and their invasive range in Hawaii in 2008 (Table S1, Supporting information). Native samples were collected from 170 traps within the states of California, Colorado, Oregon, Wyoming, and New Mexico. Invasive samples were collected from 178 traps from the Hawaiian Islands of Kauai, Oahu, Molokai, Lanai, Maui, and Hawaii (Fig. 1). Specimens were collected by deploying 5–15 Seabright Yellow Jacket and Wasp Traps^®^, separated by ≥325 m, for 24–48 h. The traps were baited with n‐heptyl butyrate emitted from controlled‐release dispensers (Landolt et al. 2003). Wasps collected in traps were placed in 95% ethanol for subsequent genetic analysis.

Sampling was conducted in a hierarchical manner consisting of four levels: traps, transects, regions, and ranges. Multiple traps were set along more or less linear transects, which spanned up to 14.5 km. Several transects were found within regions, defined as either the focal state in the native habitat or island in the invasive habitat. Regions were then grouped into two distinct ranges; the native range consisted of all states in the mainland of the United States and the invasive range consisted of the Hawaiian Islands (Table 1).

**Table 1:** Total numbers of traps and individual *Vespula pensylvanica* wasps collected from each transect in the different sampled ranges and regions

Range	Region	Transect	Traps	Individuals
Native	California	Atascandero	8	38
		Balboa Park	5	21
		BR	8	29
		Corning	3	8
		Diablo	10	43
		La Jolla	2	9
		Lake Shasta	7	27
		Los Padres	9	43
		Morgan Hill	4	20
		Portrero Road	7	23
		Ramona	11	43
		Santa Maria	8	31
		Tilden Park	13	59
		Tres Pinos	8	29
	Colorado	Loveland	2	10
		Outside Fort Collins	4	20
		Outside Larimer County	1	5
		Within Fort Collins	8	35
	New Mexico	Chimayo	1	5
	Oregon	Chemult	1	2
		Columbia River Gorge	3	7
		Klamath Falls	1	4
		Mill City	4	8
		Salem Area	37	131
		Sisters	3	5
	Wyoming	Chugadul Caspar	2	5
Invasive	Hawaii	Kahuku	11	45
		SRA	7	22
	Kauai	Highway‐552	11	35
		Makaha Ridge	9	40
	Lanai	Garden of the Gods	1	5
		Monroe Trail	23	101
		Shipwreck	1	1
	Maui	Hosmer Grove	4	4
		Haleakala	7	25
		Maui Iao Valley	10	45
		Olinda Road	13	62
		Waihee Ridge Trail	14	64
		Waipoli Road	9	30
	Molokai	Forest Reserve Road	27	122
		Molakai Kalaupapa 23	2	8
	Oahu	Manana	9	33
		Satellite Road	11	39
		WV	9	23
Total			348	1364

### DNA extraction and genotyping

We assayed the multilocus genotype of 1364 *V. pensylvanica* workers at the following 15 microsatellite markers: VMA6, LIST2002, LIST2003, LIST2004, LIST2007, LIST2008, LIST2010, LIST2014, LIST2015, LIST2017, LIST2019, LIST2020, RUFA3, RUFA5, and RUFA19 (Thoren et al. 1995; Daly et al. 2002; Hasegawa and Takahashi 2002). DNA was extracted from the legs of *V. pensylvanica* workers using a modified Chelex protocol (Goodisman et al. 2001). Loci were PCR‐amplified with fluorescently labeled primers (Hoffman et al. 2008). The resulting PCR fragments were subsequently run on an ABI 3100 Genetic Analyzer. Alleles were scored using GeneMarker v 4 (SoftGenetics).

### Genetic data analysis

Genetic diversity measures, including number of alleles, effective number of alleles, observed heterozygosity (*H*_o_), and expected heterozygosity (*H*_e_), were calculated with GenAlEx v 6.5 (Peakall and Smouse 2012). We used GENEPOP v 4.3 to test for deviations of genotype frequencies from Hardy–Weinberg equilibrium within each transect (Rousset 2008). The Bonferroni correction was used to adjust for multiple testing.

Our initial analysis detected significant deviations from Hardy–Weinberg equilibrium in 32 transects. We found that most departures were caused by an excess of homozygosity at the locus LIST2002. Microchecker v 2.2 was thus used to detect the presence of null alleles by identifying heterozygote deficiencies at each locus (van Oosterhout et al. 2006). We confirmed that LIST2002 displayed significant evidence of null alleles in 26 of 44 transects. Due to the deviation from Hardy–Weinberg equilibrium and the putative presence of null alleles, we eliminated LIST2002 from our analyses. All subsequent statistical tests were thus performed without LIST2002.

We screened for linkage disequilibrium between loci within transects using GENEPOP v 4.3 (Rousset 2008). Default parameters were used for all tests. Allele number and sample size corrected allelic richness were calculated with FSTAT v 2.9.3 (Goudet 1995). Wilcoxon signed‐rank tests were used to compare allelic richness between the native and invasive ranges, and also among regions within ranges. We used Friedman tests to compare levels of allelic richness among the Hawaiian Islands in the invasive range.

We estimated Weir and Cockerham's ϴ using Genetic Data Analysis (GDA) v 1.1 in order to assess levels of genetic differentiation (Weir and Cockerham 1984; Weir 1996; Holsinger and Weir 2009). Estimates of population structure were obtained at multiple levels, including individuals within traps, traps within transects, transects within regions, and regions within ranges. GDA was also used to calculate 95% confidence intervals (based on 1000 bootstraps) around estimates of ϴ. Traps, transects, and regions with less than two samples were excluded from the analyses.

Pairwise measures of *F*_ST_ were calculated between all traps using GENEPOP. These measures of genetic distance were then compared to geographic distance to determine whether *V. pensylvanica* displayed evidence of genetic isolation by distance (Wright 1943; Rousset 2008). The significance of the correlation between geographic and genetic distance was assessed with Mantel tests. These tests were performed with 1000 permutations in the R package vegan v 2.0 (Dixon 2003).

Individuals were assigned to putative populations using Bayesian clustering as implemented by STRUCTURE v 2.2 (Pritchard et al. 2000). To estimate the number of populations (*K*) present in the native range and invasive range, we performed different simulations, each under the assumption of a different *K* value (1 to 44), representing the total number of transects. For each simulation, we used an admixture model with uncorrelated allele frequencies to account for wasps with mixed ancestry and the LOCPRIOR model to use sampling location to inform clustering. Each simulation was run 10 times with 10,000 steps of burn‐in and 50,000 Markov chain Monte Carlo (MCMC) repetitions. The most likely number for *K* was selected based on log likelihood and the ΔK statistic developed by Evanno et al. (2005) as implemented in STRUCTURE HARVESTER (Pritchard et al. 2000; Evanno et al. 2005; Earl and Vonholdt 2012). For a given set of simulations for each *K*, CLUMPP v 1.1.2 was used to align the 10 replicate runs (Jakobsson and Rosenberg 2007). Distruct v 1.1 was then used to visualize the results of the clustering process (Rosenberg 2004).

We used the program GeneClass2 to determine the origin of individuals from the invasive range (Piry et al. 2004). We first used assignment tests to determine the likelihood that an individual *V. pensylvanica* wasp in the invasive range was from an identified reference population in native range. For the assignment tests, we used the Bayesian criteria developed by Rannala and Mountain (1997) with an assignment threshold of 0.05. We also used GeneClass2 to exclude native regions as source populations for invasive *V. pensylvanica*. To exclude individuals, we used the Bayesian criteria from Rannala and Mountain (1997) along with the resampling algorithm from Paetkau et al. (2004). We calculated the exclusion probability for each introduced individual with 1000 MCMC simulations and an alpha level of 0.01.

We used the program DIYABC 2.0 (Cornuet et al. 2014) to further understand the invasion process and detect possible source populations of invasive *V. pensylvanica*. DIYABC 2.0 uses approximate Bayesian computation (ABC) which is a Bayesian approach that compares the posterior probabilities of a large number of simulated datasets under given models to those calculated from observed data (Beaumont 2010). For each test, we compared scenarios to find potential source populations of an invasive population and to check for the presence of low effective population size after introduction (bottleneck). We compared four scenarios: (1) the focal invasive population was sourced from the native western regions (CA/OR), (2) the focal invasive population was sourced from the native central regions (WY/CO/NM), (3) the focal invasive population was sourced from the native western regions and underwent a bottleneck, and (4) the focal invasive population was sourced from the native central regions and underwent a bottleneck. The models included uniform priors with the following constraints: t2 > t1, db < t2, and N1b < N1. A generalized stepwise mutation model was used for all analyses. Each test generated reference tables with 4 × 10^5^ simulated datasets. Posterior probabilities were estimated for each scenario using polychotomous logistic regression.

Poptree2 was used to generate neighbor‐joining (NJ) trees for individuals within transects and regions (Takezaki et al. 2010). Each tree was constructed using Nei's D_A_ distance. Node confidence was assessed using 1000 bootstraps (Nei et al. 1983).

Finally, the program Bottleneck was used to identify populations that may have recently undergone a decrease in population size (Luikart et al. 1998; Piry et al. 1999). This program exploits the principle that allele number is reduced faster than heterozygosity in populations that have recently experienced a reduction in effective population size. We used the Wilcoxon test with the two‐phase mutation model (TPM), which is recommended for microsatellite datasets with small sample sizes per population and low numbers of polymorphic loci, to determine whether populations showed significant evidence of having passed through a recent bottleneck.

## Results

### Genetic diversity

We investigated whether levels of genetic diversity differed between native and invasive *V. pensylvanica*. We found that wasps from the native range had significantly higher allelic richness (Wilcoxon Sign‐Rank Test; *P *<* *0.001) and effective number of alleles (*P *=* *0.001) than wasps from the invasive range (Table 2). We also found that number of private alleles in the native range (30 total) differed significantly (*P *=* *0.0219) from the number of private alleles in the invasive range (8 total). Finally, the native range had a significantly higher level of expected heterozygosity (*P *=* *0.001) and observed heterozygosity (*P *=* *0.023) when compared to the invasive range. Overall, these results suggest that there is a slightly, but significantly, higher level of genetic diversity in the native range than the invasive range of *V. pensylvanica*.

**Table 2:** Measures of genetic diversity at microsatellite loci for native and invasive *Vespula pensylvanica*, including number of alleles (*N*_a_), effective number of alleles (*N*_e_), observed heterozygosity (*H*_o_), expected heterozygosity (*H*_e_), allelic richness (*A*), and number of private alleles (*N*_p_)

Locus	Range	*N*_a_	*N*_e_	*H*_o_	*H*_e_	*A*	*N*_p_
LIST2003	Native	17	3.815	0.719	0.738	16.515	7
	Invasive	11	3.293	0.615	0.696	10.773	1
LIST2004	Native	10	6.081	0.848	0.836	9.996	2
	Invasive	8	5.268	0.721	0.810	8.000	0
LIST2007	Native	19	8.993	0.857	0.889	18.656	6
	Invasive	13	7.195	0.701	0.861	12.800	0
LIST2008	Native	11	3.619	0.704	0.724	10.807	5
	Invasive	7	3.766	0.658	0.734	6.798	0
LIST2010	Native	17	8.449	0.852	0.882	16.611	7
	Invasive	10	6.005	0.694	0.833	10.000	0
LIST2014	Native	26	4.348	0.701	0.770	25.735	11
	Invasive	15	4.841	0.697	0.793	14.954	0
LIST2015	Native	9	4.380	0.726	0.772	8.971	1
	Invasive	9	3.766	0.605	0.734	8.576	1
LIST2017	Native	9	1.681	0.382	0.405	8.631	3
	Invasive	6	1.827	0.415	0.453	5.792	0
LIST2019	Native	6	1.671	0.390	0.402	6.000	1
	Invasive	7	2.101	0.521	0.524	6.588	2
LIST2020	Native	25	10.593	0.907	0.906	24.301	10
	Invasive	15	6.518	0.770	0.847	14.911	0
RUFA19	Native	16	5.726	0.833	0.825	15.997	2
	Invasive	14	3.833	0.674	0.739	13.752	0
RUFA3	Native	30	7.757	0.632	0.871	30.000	13
	Invasive	21	5.032	0.546	0.801	20.683	4
RUFA5	Native	18	6.472	0.821	0.846	17.938	9
	Invasive	10	5.289	0.632	0.811	9.446	1
VMA6	Native	28	12.892	0.889	0.922	27.764	7
	Invasive	22	7.528	0.748	0.867	21.453	1
Mean for all loci	Native	17.214	6.177	0.733	0.771	16.994	6.000
	Invasive	12.000	4.733	0.643	0.750	11.752	0.714

We next investigated differences in genetic diversity between different islands in the invasive Hawaiian range (Table S2, Supporting information). There were significant differences in observed heterozygosity (Friedman test; *P *=* *0.0023), expected heterozygosity (*P *<* *0.001), effective number of alleles (*P *<* *0.001), and number of private alleles (*P *<* *0.001) among the islands. Interestingly, the island of Hawaii had the highest effective number of alleles, observed heterozygosity, expected heterozygosity, and allelic richness. Maui had highest number of private alleles. In contrast, Kauai had the lowest observed heterozygosity, expected heterozygosity, allelic richness, effective number of alleles, and number of private alleles.

Recently bottlenecked populations may display an excess of heterozygosity compared to expected heterozygosity calculated from observed allele number (Cornuet and Luikart 1996; Luikart et al. 1998). We found significant excesses of heterozygosity in Maui (*P *=* *0.034) and New Mexico (*P *<* *0.001). Additionally, there were marginally significant excesses of heterozygosity present in the islands of Hawaii (*P *=* *0.052), Lanai (*P *=* *0.052), and Molokai (*P *=* *0.086). In contrast, Oahu (*P *=* *0.852) and Kauai (*P *=* *0.380) displayed no signs of bottlenecks. Overall, there is some evidence for population bottlenecks in the eastern islands of Hawaii but not the western islands.

### Genetic differentiation

We measured genetic differentiation among hierarchically structured traps, transects, regions, and ranges of *V. pensylvanica*. We first considered measures of genetic structure for all individuals, combining data from both the native and invasive ranges. The metric *f*, which measures true inbreeding within populations, was relatively low, albeit significant (Table 3). We also uncovered significant differentiation at higher levels of sampling structure. Differentiation between transects within regions, as well as regions within ranges, was moderate. In spite of these results, we found no significant genetic differentiation between the native and invasive ranges (Table 3).

**Table 3:** *F*‐statistics (and upper and lower bounds of 95% confidence intervals) for different levels of genetic structure in *Vespula pensylvanica*

	All samples	Native range	Invasive range
*f*	0.029 (0.004, 0.07)	0.012 (−0.020, 0.065)	0.001 (−0.018, 0.041)
*F*	0.112 (0.082, 0.156)	0.049 (0.017, 0.098)	0.167 (0.139, 0.224)
ϴ_traps_	–	0.037 (0.031, 0.043)	0.167 (0.147, 0.196)
ϴ_transects_	0.085 (0.075, 0.098)	0.011 (0.009, 0.013)	0.157 (0.133, 0.184)
ϴ_regions_	0.036 (0.030, 0.042)	0.003 (0.001, 0.005)	0.137 (0.111, 0.164)
ϴ_ranges_	−0.003 (−0.008, 0.003)	–	–

We next assessed the level of genetic differentiation between different hierarchical levels within the native and invasive ranges separately. We found significant genetic differentiation at most hierarchical levels in both ranges, although there were substantial differences in the magnitudes of differentiation in the native and invasive habitats. In the native range, measures of ϴ were relatively low (Table 3). In contrast, all measures of ϴ for the invasive range were high and strongly significant (Table 3). Overall, there was substantially more genetic differentiation across hierarchical levels in the invasive range than the native range.

Pairwise *F*_ST_ values were calculated between all regions (Table 4). Values of *F*_ST_ were often less than 0.01 for comparisons within the native region, indicating relatively low levels of differentiation. In contrast, values of *F*_ST_ in the invasive range were substantially higher, with many estimates exceeding 0.1. In addition, all pairwise comparisons involving Kauai had *F*_ST_ values greater than 0.2, suggesting that Kauai may be the most genetically distinct island in the invasive range.

**Table 4:** Pairwise estimates of *F*_ST_ for all regions

	California	Colorado	Oregon	Wyoming	New Mexico	Molokai	Hawaii	Kauai	Lanai	Maui
Colorado	0.009^a^									
Oregon	0.005^a^	0.008^a^								
Wyoming	0.019^a^	0.005	0.014							
New Mexico	0.029^a^	0.026^a^	0.034^a^	0.031^a^						
Molokai	0.083^a^	0.095^a^	0.081^a^	0.111^a^	0.107^a^					
Hawaii	0.033^a^	0.046^a^	0.034^a^	0.054^a^	0.072^a^	0.124^a^				
Kauai	0.171^a^	0.197^a^	0.168^a^	0.244^a^	0.277^a^	0.244^a^	0.222^a^			
Lanai	0.091^a^	0.101^a^	0.095^a^	0.122^a^	0.101^a^	0.095^a^	0.118^a^	0.279^a^		
Maui	0.049^a^	0.059^a^	0.053^a^	0.076^a^	0.074^a^	0.119^a^	0.084^a^	0.230^a^	0.067^a^	
Oahu	0.064^a^	0.084^a^	0.066^a^	0.108^a^	0.109^a^	0.096^a^	0.108^a^	0.218^a^	0.135^a^	0.111^a^

a *P* < 0.05.

**Figure 2:**
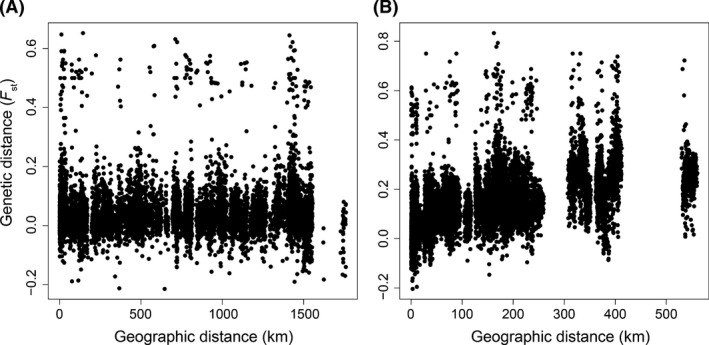
Relationship between genetic distance (*F*_ST_) and geographic distance (km) in the (A) native mainland (Mantel test *r* = 0.042, *P* = 0.102) and (B) invasive Hawaiian range (*r* = 0.569, *P* < 0.001) of *Vespula pensylvanica*.

We tested for the presence of genetic isolation by distance. Our analysis revealed no significant correlation between genetic distance (*F*_ST_) and geographic distance (km) for individuals sampled from different traps in the native range (Mantel test *r* = 0.042, *P *=* *0.102) (Fig. 2A). In contrast, there was a strong and significant isolation‐by‐distance relationship between individuals sampled from different traps within the invasive region (*r* = 0.569, *P *<* *0.001) (Fig. 2B). We found no evidence for isolation by distance within the individual Hawaiian Islands of Kauai (*r* = −0.011, *P *=* *0.546), Oahu (*r* = 0.062, *P *=* *0.181), Molokai (*r* = −0.028, *P *=* *0.665), or Lanai (*r* = 0.120, *P *=* *0.157). However, we did find significant isolation‐by‐distance relationships in Hawaii (*r* = 0.163, *P *=* *0.005) and Maui (*r* = 0.063, *P *=* *0.002).

### Genetic clustering

**Figure 3:**
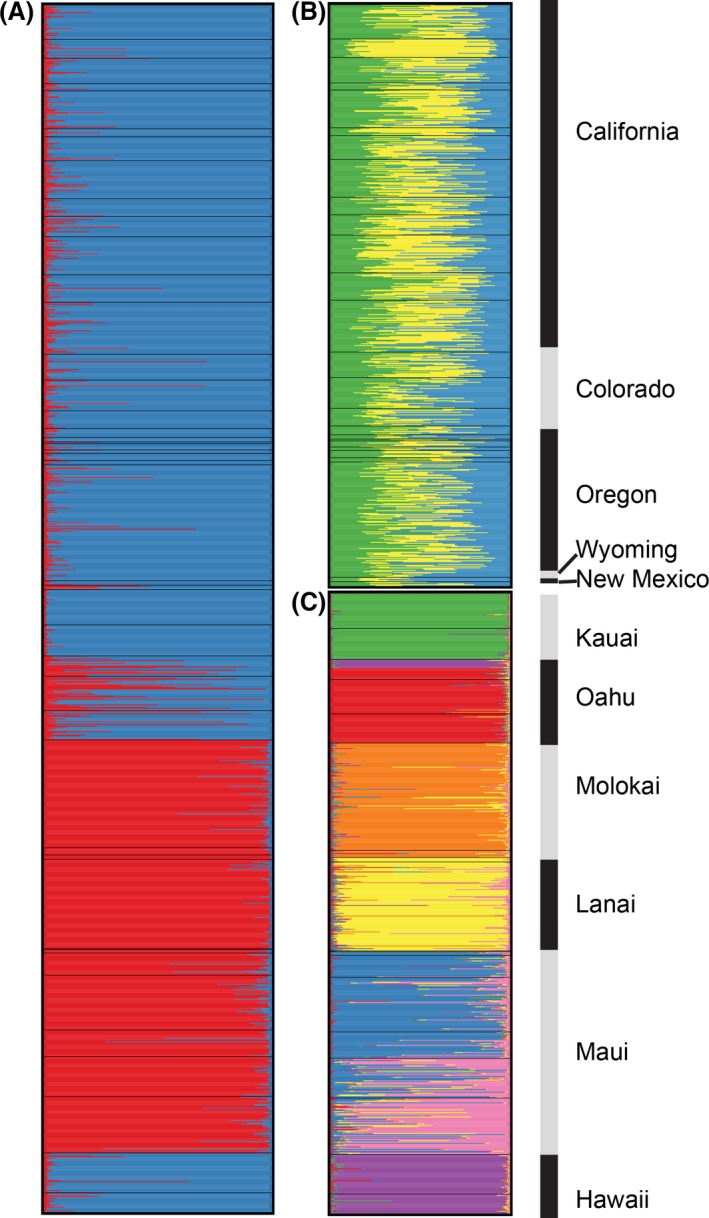
Estimated membership coefficients for individuals in each of *K* putative populations in the (A) combined native and invasive ranges (*K* = 2), (B) native range only (*K* = 3), and (C) invasive range only (*K* = 7). All transects in Hawaii are ordered west to east starting at the top with the island of Kauai. Each line represents an individual, the color of which corresponds to the estimated membership of that individual in a certain cluster; the same colors are used to represent different populations in the different figure panels. Sample origin is denoted by gray and black bars.

*Vespula pensylvanica* wasps from the native and invasive ranges were both clustered into putative populations based on their multilocus genotypes. This analysis grouped individuals into two genetically distinct populations (Fig. 3A). All of the individuals from the native range formed a single population. Individuals from the islands of Hawaii, Kauai, and Oahu in the invasive regions clustered into this single population. Conversely, individuals from Molokai, Lanai, and Maui formed another distinct population separate from the other island and the mainland regions. Most individuals were assigned to a single cluster, suggesting a lack of admixture.

We performed the clustering analysis considering only individuals from the native range. In this case, it was difficult to assign the most likely number of populations (*K*), as the Δ*K* metric was similar for *K* = 2 and 3. Nevertheless, *K* = 3 had the highest Δ*K*. Interestingly, almost all individuals in the native range were partially assigned to all three putative populations (Fig. 3B). Individuals sampled from Balboa Park transect in California were a slight exception and tended to form a more distinct group than individuals sampled from other transects. However, the overall analysis indicated a general lack of genetic structure within the native range of *V. pensylvanica*.

We next clustered individuals within the invasive range only. The Δ*K* metric produced a clear peak, in contrast to the analysis of the native range samples, suggesting the most likely number of populations was seven (Fig. 3C). Individuals within islands tended to form distinct clusters. Specifically, *V. pensylvanica* within Molokai, Kauai, and Lanai each formed separate and distinct populations. Samples from Oahu were separated into two clusters, where individuals were either part of a cluster that also consisted of samples from Hawaii or part of a cluster that consisted solely of samples from Oahu (Fig. 3C). All of the Oahu individuals that clustered with Hawaiian samples were collected from a single transect, Satellite Road, and showed no signs of admixture. In contrast, individuals from Maui displayed signs of admixture. Most individuals from Maui were partially assigned to two clusters; however, the fractional memberships of individuals varied by transect. Individuals from the transects of Hosmer Grove, Haleakala, Olinda Road, and Waipoli Road had a higher probability of being assigned to one cluster (Fig. 3C) while individuals from Maui Iao Valley and Waihee Ridge Trail had a higher probability of being assigned to the other Maui cluster (Fig. 3C). Regardless, the overall analysis of samples from the invasive range showed substantial evidence for population genetic structure both within and between islands.

We assigned individuals from the invasive range to regions in the native range to determine the potential origins of invasive *V. pensylvanica* in Hawaii. We found that 80% of the individuals from the invasive range had the highest score of being assigned to the western regions of the native range (Table S3A, Supporting information). Overall, the mean assignment scores for the western regions were higher than those found for the central regions (Table 5a). This suggested that the western part of the native range was the most probable source of the invasive population. Notably, however, exclusion probabilities were generally high for all individuals (*P* > 0.05), suggesting that we cannot exclude either the western or the central regions as the source population for invasive *V. pensylvanica* (Table S3B, Supporting information*)*.

**Table 5:** Assignment of invasive *Vespula pensylvanica* populations to the western regions (California and Oregon) or central regions (Wyoming, Colorado, and New Mexico) of the native range. (a) Assignment scores of individuals from invasive regions to combined reference regions. (b) Relative posterior probability (with 95% C.I. in parentheses) for demographic scenarios where invasive regions were derived from either the western or central regions with or without associated bottlenecks

	a. Scores		b. Relative posterior probability			
Island	West	Central	West, no bottleneck	Central, no bottleneck	West, bottleneck	Central, bottleneck
Kauai	90.2	9.8	0.28 (0.149–0.410)	0.504 (0.410–0.598)	0.07 (0.000–0.173)	0.146 (0.066–0.226)
Molokai	67.5	32.5	0.06 (0.038–0.082)	0.735 (0.683–0.786)	0.01 (0.000–0.022)	0.195 (0.148–0.243)
Maui	76.2	23.8	0.142 (0.095–0.190)	0.706 (0.648–0.764)	0.017 (0.008–0.026)	0.134 (0.098–0.171)
Lanai	70.2	29.8	0.142 (0.100–0.185)	0.726 (0.673–0.779)	0.019 (0.010–0.027)	0.113 (0.082–0.143)
Hawaii	87.6	12.4	0.27 (0.210–0.329)	0.575 (0.515–0.635)	0.031 (0.012–0.050)	0.125 (0.091–0.158)
Oahu	90.9	9.1	0.122 (0.085–0.160)	0.78 (0.733–0.825)	0.018 (0.011–0.024)	0.081 (0.058–0.105)
All Islands	78.2	21.8	0.283 (0.194–0.371)	0.307 (0.217–0.398)	0.199 (0.120–0.277)	0.211 (0.131–0.291)

In contrast to the assignment tests, approximate Bayesian computation suggested that the central regions of the native range were the most likely source of the invasive populations (Table 5b). Specifically, the scenario where individuals from the invasive range were derived from the central part of the native range without a bottleneck had the highest probability among different tested scenarios (Posterior Probability = 0.655; 95% C.I. of 0.603 to 0.708).

**Figure 4:**
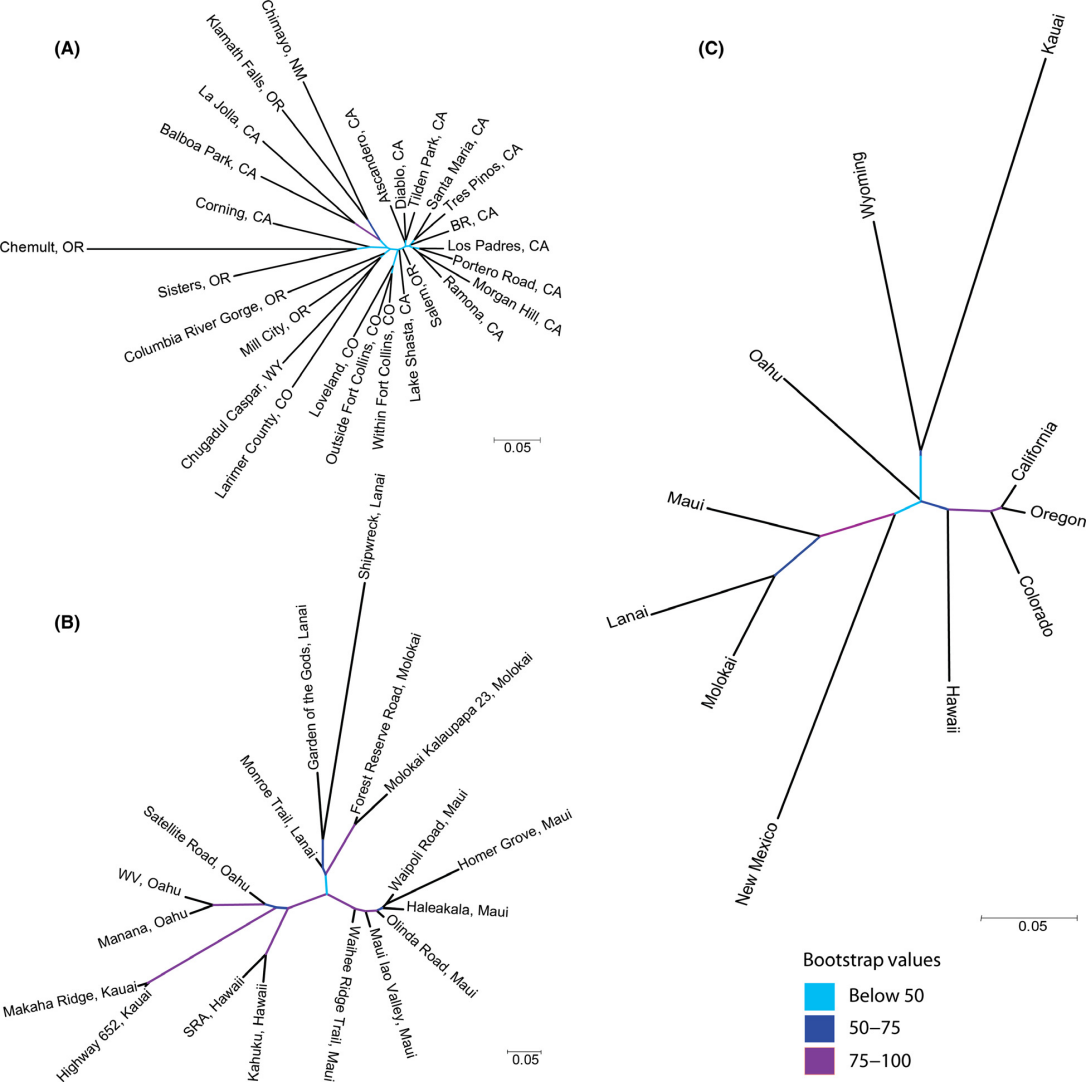
Unrooted neighbor‐joining trees for (A) native transects only (B) invasive transects only, and (C) all regions. Bootstrap support for nodes is represented by color.

We visualized the relationships between individuals sampled from different transects with neighbor‐joining trees. Transects from the native regions formed a starlike structure, indicating a lack of strong genetic relationships in the native range (Fig. 4A). In contrast, transects in the invasive range from the same island clustered together, reflecting the genetic differences between islands (Fig. 4B). We also produced a neighbor‐joining tree of all regions in both the native and invasive ranges and found that samples from Maui, Lanai, and Molokai formed a single group while samples from Hawaii, Oahu, and Kauai grouped with mainland regions (Fig. 4C).

## Discussion

### Small reduction in genetic diversity in the invasive range of *V. pensylvanica*

Introduced species tend to experience drops in genetic diversity due to population bottlenecks derived from founder events (Luikart et al. 1998; Goodisman et al. 2001; Sakai et al. 2001; Dlugosch and Parker 2008). Reduced genetic diversity could have negative effects on invasion success because it can affect a population's growth and ability to adapt to changing selection pressures (Sakai et al. 2001). However, a lack of genetic diversity does not necessarily preclude population growth or adaptation (Dlugosch and Parker 2008; Moran and Alexander 2014). Thus, there is considerable interest in understanding whether invasive species experience losses of genetic diversity and whether such losses are associated with invasion success (Dlugosch and Parker 2008; Purcell et al. 2012; Moran and Alexander 2014).

We compared levels of genetic diversity found within the native and invasive ranges of *V. pensylvanica*. Overall, greater levels of genetic diversity were observed in the native range than the invasive range (Table 2). However, the differences in variation between the two ranges were modest. *Vespula pensylvanica* from the invasive range had 97% of the expected heterozygosity and 64% of the allelic richness found in the native range. The overall drop in expected heterozygosity is quite small compared to the drop in allelic richness, which is a characteristic of a brief population bottleneck (Luikart et al. 1998). In this case, some rare alleles are lost, although observed heterozygosity, which is more strongly influenced by common alleles, is not severely reduced (Luikart et al. 1998).

The allelic richness lost by invasive *V. pensylvanica* is similar to that lost by some other invasive social insects, such as the Formosan subterranean termite, *Coptotermes formosanus*, and the paper wasp, *Polistes chinensis antennalis*, in their invasive ranges (Husseneder et al. 2012; Tsuchida et al. 2014). In contrast, the Argentine ant, *Linepithema humile*, the Eastern Subterranean termite, *Reticulitermes flavipes*, and the Buff‐tailed bumblebee, *Bombus terrestris*, experienced drops in allelic richness of 50% or more in their invasive ranges (Tsutsui et al. 2000; Vargo 2003; Schmid‐Hempel et al. 2007).

In addition to a reduction of genetic diversity in the invasive range, we detected some evidence of genetic bottlenecks in the eastern islands of Molokai, Maui, Hawaii, and Lanai. Interestingly, there was no significant evidence for bottlenecks in Kauai and Oahu. The populations on both of these islands were introduced in the early 1900s (Nakahara 1980), so it is possible that allelic diversity and heterozygosity may have reached equilibrium, making it difficult to detect bottlenecks (Cornuet and Luikart 1996; Luikart et al. 1998). In contrast, populations on Molokai, Maui, Hawaii, and Lanai were established more recently and may not have reached equilibrium with respect to allelic heterozygosity.

In other invasive social insects, losses of genetic diversity have been implicated in the development of supercolonies, which are large, multiqueen colonies that consist of multiple nests and lack substantial boundaries (Holway et al. 2002; Tsutsui and Suarez 2003; Suarez and Tsutsui 2008; Helantera et al. 2009). Hanna et al. (2014a) showed that workers from native colonies of *V. pensylvanica* were always produced by a single queen, whereas colonies in the invasive range often contained workers produced by multiple queens (Goodisman et al. 2001; Hanna et al. 2014a). It is possible that the reduction of genetic diversity found in invasive *V. pensylvanica* is associated with this change in social structure and invasion success. However, the magnitude of genetic diversity in the invasive range is still high compared to other introduced species that produce supercolonies (Helantera et al. 2009). In addition, perennial *V. pensylvanica* colonies can be found in parts of the native range, suggesting that phenotypic plasticity, rather than changes in genetic diversity, might be involved in the formation of multiqueen, perennial *Vespula* nests (Gambino 1991; Visscher and Vetter 2003).

### Lack of genetic structure in the native range of *V. pensylvanica*

*Vespula pensylvanica* showed a remarkable lack of genetic structure in its native range in the United States, which stands contrary to our original prediction (Table 3). There was little evidence for genetic differentiation among hierarchically sampled locations and no significant evidence of genetic isolation by distance, suggesting a high level of gene flow across the entire native range (Fig. 2A and Table 3). This is particularly notable because our sampling scheme spanned over 2000 km of western North America. The lack of genetic structure in the native range of *V. pensylvanica* could have resulted from human‐mediated dispersal, which may have led to high rates of gene flow across the native range (Moller 1996). Alternatively, the dispersal distances of *Vespula* queens may be sufficient to develop genetic homogeneity over long, evolutionary timescales.

Our finding that native *V. pensylvanica* lacks genetic structure parallels results found in other native *Vespula* species over smaller ranges. For example, Hoffman et al. (2008) failed to detect genetic structure in *Vespula maculifrons* and *Vespula squamosa* along a span of approximately 130 km in its native range of North America (Hoffman et al. 2008). The sampling range for *V. maculifrons* and *V. squamosa* was more than an order of magnitude smaller than that of *V. pensylvanica* in this study, yet the results showing genetic homogeneity of *Vespula* species in their native ranges are consistent in these studies. However, the patterns seen in *Vespula* species are contrary to those found in other invasive Hymenoptera, which tend to display more population structure in their native range than their invasive range (Auger‐Rozenberg et al. 2012; Tsuchida et al. 2014).

### The invasion history of *V. pensylvanica* in Hawaii

We attempted to identify potential source populations of invasive *V. pensylvanica* in Hawaii. Given historical records, we expected that Oregon would be the source of invasive populations (Nakahara 1980). In accord with this prediction, we found that a majority of introduced individuals were assigned to the western part of the native range using one particular assignment test (Table 5A). However, a different assignment procedure suggested that the central area of the native range was the most likely source of invasive *V. pensylvanica* (Table 5B). These contrasting results may reflect the general lack of genetic differentiation among native *V. pensylvanica* populations, which may make the determination of the source of invasive populations difficult to ascertain. In addition, our limited sampling from the central regions of native *V. pensylvanica* may preclude our ability to assign source populations with high confidence (Muirhead et al. 2008).

*Vespula pensylvanica* displayed substantial levels of genetic differentiation both between and within Hawaiian Islands, in contrast to our expectations that genetic differentiation would be limited. For example, we found that the relationship between genetic and geographic distance in the invasive range was nonlinear and displayed gaps at certain spans of geographic distance (Fig. 2B). This trend mostly reflected strong genetic differences between islands combined with modest genetic structure within islands. The difference in patterns of isolation by distance between the native and invasive ranges was particularly notable given the great difference in geographic distance in the ranges. The native range stretches across 2000 km of western United States, while the invasive range spans approximately 600 km. Yet the invasive range showed substantially greater levels of genetic structure and isolation by distance. These trends could result, in part, from the expanses of ocean between the Hawaiian Islands (Pierce et al. 2014). The differences between native and introduced species may also reflect nonequilibrium conditions found in the introduced range (Akre et al. 1981). A similar, significant isolation‐by‐distance relationship, spanning approximately 225 km, was found for *V. germanica* in its invasive region of Australia (Goodisman et al. 2001).

We also found significant differences in the levels of genetic diversity among the Hawaiian Islands. Out of all the islands, Kauai had the lowest levels of all diversity metrics (Table 2). A survey conducted by the Hawaii Department of Agriculture suggests that *V. pensylvanica* was introduced multiple times to Kauai (Nakahara 1980). Multiple introductions are generally expected to result in greater genetic variation in an invasive habitat (Sakai et al. 2001; Kolbe et al. 2004). Therefore, it was somewhat unexpected that Kauai would have low levels of genetic variation. Kauai was also the most genetically distinct population when compared to other islands in the invasive range.

The *V. pensylvanica* population on Oahu was also thought to have been founded by multiple introductions (Nakahara 1980). Levels of genetic diversity in Oahu were relatively high and individuals within Oahu formed two distinct populations (Fig. 3A). These results suggest that *V. pensylvanica* may have been introduced multiple times to Oahu. This island contains a large percent of the Hawaiian Island's human population, so it is possible that such putative introductions were facilitated by human‐mediated shipments from the mainland (Nakahara 1980).

The existence of a discrete genetic population consisting of all individuals from Hawaii and a few individuals from Oahu was surprising as Oahu and Hawaii are separated by the islands of Molokai, Lanai, and Maui (Figs. 1, 3C). It thus seems unlikely that Oahu individuals directly seeded the Hawaiian population, or vice versa, through natural dispersal. However, human‐mediated dispersal could account for this pattern. Regardless, all of the aberrant Oahu individuals originated from a single transect that was the most western of all the Oahu transects. As these individuals were confined to the western section of the island, physical barriers, such as the volcanoes Wai'anae or Ko'olau, may have prevented gene flow and the homogenization of allele frequencies across Oahu (Roderick and Gillespie 1998).

The islands of Molokai and Lanai also each formed a separate, genetically distinct population from all of the other islands (Fig. 3C). This is consistent with the idea that Molokai and Lanai were colonized through single, separate introductions in the late 1970s (Nakahara 1980). Even though both islands formed a distinct population, Molokai and Lanai cluster together in the NJ tree (Fig. 4B). The populations on both islands may have been seeded by genetically similar source populations. Substantial genetic drift may have occurred during population formation, creating genetically distinct populations on each island.

Finally, we uncovered an unusual pattern of genetic structure in Maui. Individuals from transects on the eastern part of Maui tended to form a somewhat differentiated population from those on the western part of the island (Fig. 3C). This suggests low levels of gene flow between western and eastern *V. pensylvanica* in Maui, possibly to due to physical isolation (Roderick and Gillespie 1998). Such a result also raises the possibility that there may have been at least two introductions of *V. pensylvanica* to this island.

## Conclusions

We examined the population genetic structure of *Vespula pensylvanica*, a wasp introduced to the archipelago of Hawaii from its native range in North America (Nakahara 1980; Gambino and Loope 1992). Remarkably, we found that invasive populations displayed substantially higher levels of genetic structure than native populations. Thus, *V. pensylvanica* is capable of high levels of dispersal and gene flow, likely through human‐mediated transportation. However, such gene flow is apparently constrained in the invasive habitats of Hawaii, which consists of islands separated by wide expanses of water. The presence of genetic structure in invasive populations reflects the influence of geographic barriers, invasion dynamics, and a nonequilibrium state of population structure. Continued study of this taxon over the coming decades may be particularly useful for understanding how invasive species come to be established in their introduced habitats. Overall, study of the invasion of *V. pensylvanica* in Hawaii may provide further insight on the process of biological invasions on archipelagos, which could help in the development of policy that can prevent and curb invasions to vulnerable regions.

## Data accessibility

Sampling information and microsatellite genotypes can be found in Table S1 and S4, respectively.

## Conflict of Interest

None declared.

## Supporting information


**Table S1.** Locations and total numbers of *V. pensylvanica* wasps collected from traps in the sampled transects, ranges, and regions (NA = location not determined).
**Table S2**. Measures of genetic diversity at microsatellite loci for *V. pensylvanica* from invasive regions, including number of samples (*N*), number of alleles (*N*_a_), effective number of alleles (*N*_e_), observed heterozygosity (*H*_o_), expected heterozygosity (*H*_e_), allelic richness (*A*), and number of private alleles (*N*_p_).
**Table S3**. Assignment and Exclusion test of invasive Hawaiian populations to mainland populations in the United States.
**Table S4**. Locations of, total numbers of, and genotypes of *V. pensylvanica* wasps collected from, each trap in the sampled transects, ranges, and regions (NA = location not determined) at 15 loci.

## References

[ece31757-bib-0001] Akre, R. D. , A. Greene , J. F. MacDonald , P. J. Landolt , and H. G. Davis . 1981 The yellowjackets of America north of Mexico. Pp. 1–102. U S Department of Agriculture Agriculture Handbook, Issue 552.

[ece31757-bib-0002] Auger‐Rozenberg, M. A. , T. Boivin , E. Magnoux , C. Courtin , A. Roques , and C. Kerdelhue . 2012 Inferences on population history of a seed chalcid wasp: invasion success despite a severe founder effect from an unexpected source population. Mol. Ecol. 21:6086–6103. 23110419 10.1111/mec.12077

[ece31757-bib-0003] Beaumont, M. A. 2010 Approximate Bayesian computation in evolution and ecology. Annu. Rev. Ecol. Evol. Syst. 41:379–406.

[ece31757-bib-0004] Beggs, J. R. , E. G. Brockerhoff , J. C. Corley , et al. 2011 Ecological effects and management of invasive alien *Vespidae* . Biocontrol 56:505–526.

[ece31757-bib-0005] Brockerhoff, E. G. , B. I. P. Barratt , J. R. Beggs , et al. 2010 Impacts of exotic invertebrates on New Zealand's indigenous species and ecosystems. N. Z. J. Ecol. 34:158–174.

[ece31757-bib-0006] Chapman, R. E. , and A. F. G. Bourke . 2001 The influence of sociality on the conservation biology of social insects. Ecol. Lett. 4:650–662.

[ece31757-bib-0007] Cornuet, J. M. , and G. Luikart . 1996 Description and power analysis of two tests for detecting recent population bottlenecks from allele frequency data. Genetics 144:2001–2014. 8978083 10.1093/genetics/144.4.2001 PMC1207747

[ece31757-bib-0008] Cornuet, J.‐M. , P. Pudlo , J. Veyssier , et al. 2014 DIYABC v2.0: a software to make approximate Bayesian computation inferences about population history using single nucleotide polymorphism, DNA sequence and microsatellite data. Bioinformatics 30:1187–1189. 10.1093/bioinformatics/btt763 24389659

[ece31757-bib-0009] Daly, D. , M. E. Archer , P. C. Watts , et al. 2002 Polymorphic microsatellite loci for eusocial wasps (Hymenoptera: Vespidae). Mol. Ecol. Notes 2:273–275.

[ece31757-bib-0010] Dixon, P. 2003 VEGAN, a package of R functions for community ecology. J. Veg. Sci. 14:927–930.

[ece31757-bib-0011] Dlugosch, K. M. , and I. M. Parker . 2008 Founding events in species invasions: genetic variation, adaptive evolution, and the role of multiple introductions. Mol. Ecol. 17:431–449. 17908213 10.1111/j.1365-294X.2007.03538.x

[ece31757-bib-0012] Drake, D. R. , C. P. H. Mulder , D. R. Towns , and C. H. Daugherty . 2002 The biology of insularity: an introduction. J. Biogeogr. 29:563–569.

[ece31757-bib-0013] Earl, D. A. , and B. M. Vonholdt . 2012 STRUCTURE HARVESTER: a website and program for visualizing STRUCTURE output and implementing the Evanno method. Conserv. Genet. Resour. 4:359–361.

[ece31757-bib-0014] Elliott, G. P. , P. R. Wilson , R. H. Taylor , and J. R. Beggs . 2010 Declines in common, widespread native birds in a mature temperate forest. Biol. Conserv. 143:2119–2126.

[ece31757-bib-0015] Evanno, G. , S. Regnaut , and J. Goudet . 2005 Detecting the number of clusters of individuals using the software structure: a simulation study. Mol. Ecol. 14:2611–2620. 15969739 10.1111/j.1365-294X.2005.02553.x

[ece31757-bib-0016] Evans, T. A. , B. T. Forschler , and J. K. Grace . 2013 Biology of invasive termites: a worldwide review. Annu. Rev. Entomol. 58:455–474. 23020620 10.1146/annurev-ento-120811-153554

[ece31757-bib-0017] Foote, D. , and H. L. Carson . 1995 Drosophila as monitors of change in Hawaiian ecosystems. Pp. 368–372. *in* LaRoe E. T., ed. Our living resources: A report to the nation on the distribution, abundancy, and health of U.S. plants, animals, and ecosystems. U.S. National Biological Service, Washington, DC.

[ece31757-bib-0018] Gambino, P. 1991 Reproductive plasticity of *Vespula pensylvanica* (Hymenoptera, Vespidae) on Maui and Hawaii Islands, USA. N. Z. J. Zool. 18:139–149.

[ece31757-bib-0019] Gambino, P. , and L. Loope . 1992 Yellowjacket (Vespula pensylvanica) biology and abatement in the National Parks of Hawaii. University of Hawaii Cooperative National Park Resources Studies Unit Technical Report, i‐v, 1‐41.

[ece31757-bib-0020] Goodisman, M. A. D. , R. W. Matthews , and R. H. Crozier . 2001 Hierarchical genetic structure of the introduced wasp *Vespula germanica* in Australia. Mol. Ecol. 10:1423–1432. 11412365 10.1046/j.1365-294x.2001.01291.x

[ece31757-bib-0021] Gotzek, D. , H. J. Axen , A. V. Suarez , S. H. Cahan , and D. Shoemaker . 2015 Global invasion history of the tropical fire ant: a stowaway on the first global trade routes. Mol. Ecol. 24:374–388. 25496038 10.1111/mec.13040

[ece31757-bib-0022] Goudet, J. 1995 FSTAT (Version 1.2): A computer program to calculate F‐statistics. J. Hered. 86:485–486.

[ece31757-bib-0023] Hanna, C. , E. D. Cook , A. R. Thompson , et al. 2014a Colony social structure in native and invasive populations of the social wasp *Vespula pensylvanica* . Biol. Invasions 16:283–294.

[ece31757-bib-0024] Hanna, C. , D. Foote , and C. Kremen . 2014b Competitive impacts of an invasive nectar thief on plant‐pollinator mutualisms. Ecology 95:1622–1632. 25039226 10.1890/13-1276.1

[ece31757-bib-0025] Hasegawa, E. , and J. Takahashi . 2002 Microsatellite loci for genetic research in the hornet *Vespa mandarinia* and related species. Mol. Ecol. Notes 2:306–308.

[ece31757-bib-0026] Helantera, H. , J. E. Strassmann , J. Carrillo , and D. C. Queller . 2009 Unicolonial ants: where do they come from, what are they and where are they going? Trends Ecol. Evol. 24:341–349. 19328589 10.1016/j.tree.2009.01.013

[ece31757-bib-0027] Hoffman, E. A. , J. L. Kovacs , and M. A. D. Goodisman . 2008 Genetic structure and breeding system in a social wasp and its social parasite. BMC Evol. Biol., 8:239. doi: 10.1186/1471‐2148‐8‐239. 18715511 10.1186/1471-2148-8-239 PMC2533669

[ece31757-bib-0028] Holsinger, K. E. , and B. S. Weir . 2009 Genetics in geographically structured populations: defining, estimating and interpreting *F* _ST_ . Nat. Rev. Genet. 10:639–650. 19687804 10.1038/nrg2611 PMC4687486

[ece31757-bib-0029] Holway, D. A. , L. Lach , A. V. Suarez , N. D. Tsutsui , and T. J. Case . 2002 The causes and consequences of ant invasions. Annu. Rev. Ecol. Syst. 33:181–233.

[ece31757-bib-0030] Husseneder, C. , D. M. Simms , J. R. Delatte , et al. 2012 Genetic diversity and colony breeding structure in native and introduced ranges of the Formosan subterranean termite, *Coptotermes formosanus* . Biol. Invasions 14:419–437.

[ece31757-bib-0031] Jakobsson, M. , and N. A. Rosenberg . 2007 CLUMPP: a cluster matching and permutation program for dealing with label switching and multimodality in analysis of population structure. Bioinformatics 23:1801–1806. 17485429 10.1093/bioinformatics/btm233

[ece31757-bib-0032] Kenis, M. , M. A. Auger‐Rozenberg , A. Roques , et al. 2009 Ecological effects of invasive alien insects. Biol. Invasions 11:21–45.

[ece31757-bib-0033] Kirk, H. , S. Dorn , and D. Mazzi . 2013 Molecular genetics and genomics generate new insights into invertebrate pest invasions. Evol. Appl. 6:842–856. 10.1111/eva.12071 PMC5779122 29387170

[ece31757-bib-0034] Kolbe, J. J. , R. E. Glor , L. R. G. Schettino , et al. 2004 Genetic variation increases during biological invasion by a Cuban lizard. Nature 431:177–181. 15356629 10.1038/nature02807

[ece31757-bib-0035] Landolt, P. J. , H. C. Reed , and D. J. Ellis . 2003 Trapping yellowjackets (Hymenoptera: Vespidae) with heptyl butyrate emitted from controlled‐release dispensers. Fla. Entomol. 86:323–328.

[ece31757-bib-0036] Luikart, G. , F. Allendorf , J.‐M. Cornuet , and W. Sherwin . 1998 Distortion of allele frequency distributions provides a test for recent population bottlenecks. J. Hered. 89:238–247. 9656466 10.1093/jhered/89.3.238

[ece31757-bib-0037] Masciocchi, M. , and J. Corley . 2013 Distribution, dispersal and spread of the invasive social wasp (*Vespula germanica*) in Argentina. Austral Ecol. 38:162–168.

[ece31757-bib-0038] Matthews, R. W. , M. A. D. Goodisman , A. D. Austin , and R. Bashford . 2000 The introduced English wasp *Vespula vulgaris* (Hymenoptera: Vespidae) newly recorded invading native forests in Tasmania. Aust. J. Entomol. 39:177–179.

[ece31757-bib-0039] Moller, H. 1996 Lessons for invasion theory from social insects. Biol. Conserv. 78:125–142.

[ece31757-bib-0040] Monceau, K. , O. Bonnard , and D. Thiery . 2014 *Vespa velutina*: a new invasive predator of honeybees in Europe. J. Pest. Sci. 87:1–16.

[ece31757-bib-0041] Moran, E. V. , and J. M. Alexander . 2014 Evolutionary responses to global change: lessons from invasive species. Ecol. Lett. 17:637–649. 24612028 10.1111/ele.12262

[ece31757-bib-0042] Muirhead, J. R. , D. K. Gray , D. W. Kelly , et al. 2008 Identifying the source of species invasions: sampling intensity vs. genetic diversity. Mol. Ecol. 17:1020–1035. 18261046 10.1111/j.1365-294X.2008.03669.x

[ece31757-bib-0043] Nakahara, L. M. 1980 Survey report on the Yellowjackets, Vespula pensylvanica and Vespula vulgaris, in Hawaii (ed. Agriculture HDo).

[ece31757-bib-0044] Nei, M. , F. Tajima , and Y. Tateno . 1983 Accuracy of estimated phylogenetic trees from molecular data. J. Mol. Evol. 19:153–170. 6571220 10.1007/BF02300753

[ece31757-bib-0045] van Oosterhout, C. , D. Weetman , and W. F. Hutchinson . 2006 Estimation and adjustment of microsatellite null alleles in nonequilibrium populations. Mol. Ecol. Notes 6:255–256.

[ece31757-bib-0046] Paetkau, D. , R. Slade , M. Burden , and A. Estoup . 2004 Genetic assignment methods for the direct, real‐time estimation of migration rate: a simulation‐based exploration of accuracy and power. Mol. Ecol. 13:55–65. 14653788 10.1046/j.1365-294x.2004.02008.x

[ece31757-bib-0047] Peakall, R. , and P. E. Smouse . 2012 GenAlEx 6.5: genetic analysis in Excel. Population genetic software for teaching and research‐an update. Bioinformatics 28:2537–2539. 22820204 10.1093/bioinformatics/bts460 PMC3463245

[ece31757-bib-0048] Pejchar, L. , and H. A. Mooney . 2009 Invasive species, ecosystem services and human well‐being. Trends Ecol. Evol. 24:497–504. 19577817 10.1016/j.tree.2009.03.016

[ece31757-bib-0049] Pierce, A. A. , M. P. Zalucki , M. Bangura , et al. 2014 Serial founder effects and genetic differentiation during worldwide range expansion of monarch butterflies. Proc. R. Soc. B 281: pii: 20142230. doi: 10.1098/rspb.2014.2230. 10.1098/rspb.2014.2230 PMC4241002 25377462

[ece31757-bib-0050] Piry, S. , G. Luikart , and J. Cornuet . 1999 BOTTLENECK: a computer program for detecting recent reductions in the effective size using allele frequency data. J. Hered. 90:502–503.

[ece31757-bib-0051] Piry, S. , A. Alapetite , J. M. Cornuet , et al. 2004 GENECLASS2: A software for genetic assignment and first‐generation migrant detection. J. Hered. 95:536–539. 15475402 10.1093/jhered/esh074

[ece31757-bib-0052] Pritchard, J. K. , M. Stephens , and P. Donnelly . 2000 Inference of population structure using multilocus genotype data. Genetics 155:945–959. 10835412 10.1093/genetics/155.2.945 PMC1461096

[ece31757-bib-0053] Purcell, K. M. , N. Ling , and C. A. Stockwell . 2012 Evaluation of the introduction history and genetic diversity of a serially introduced fish population in New Zealand. Biol. Invasions 14:2057–2065.

[ece31757-bib-0054] Rannala, B. , and J. L. Mountain . 1997 Detecting immigration by using multilocus genotypes. Proc. Natl Acad. Sci. USA 94:9197–9201. 9256459 10.1073/pnas.94.17.9197 PMC23111

[ece31757-bib-0055] Roderick, G. K. , and R. G. Gillespie . 1998 Speciation and phylogeography of Hawaiian terrestrial arthropods. Mol. Ecol. 7:519–531. 9628003 10.1046/j.1365-294x.1998.00309.x

[ece31757-bib-0056] Rosenberg, N. A. 2004 DISTRUCT: a program for the graphical display of population structure. Mol. Ecol. Notes 4:137–138.

[ece31757-bib-0057] Rousset, F. 2008 GENEPOP ‘ 007: a complete re‐implementation of the GENEPOP software for Windows and Linux. Mol. Ecol. Resour. 8:103–106. 21585727 10.1111/j.1471-8286.2007.01931.x

[ece31757-bib-0058] Sakai, A. K. , F. W. Allendorf , J. S. Holt , et al. 2001 The population biology of invasive species. Annu. Rev. Ecol. Syst. 32:305–332.

[ece31757-bib-0059] Schmid‐Hempel, P. , R. Schmid‐Hempel , P. C. Brunner , O. D. Seeman , and G. R. Allen . 2007 Invasion success of the bumblebee, *Bombus terrestris*, despite a drastic genetic bottleneck. Heredity 99:414–422. 17551520 10.1038/sj.hdy.6801017

[ece31757-bib-0060] Simberloff, D. , J. L. Martin , P. Genovesi , et al. 2013 Impacts of biological invasions: what's what and the way forward. Trends Ecol. Evol. 28:58–66. 22889499 10.1016/j.tree.2012.07.013

[ece31757-bib-0061] Smith, C. R. , A. L. Toth , A. V. Suarez , and G. E. Robinson . 2008 Genetic and genomic analyses of the division of labour in insect societies. Nat. Rev. Genet. 9:735–748. 18802413 10.1038/nrg2429

[ece31757-bib-0062] Suarez, A. V. , and T. J. Case . 2002 Bottom‐up effects on persistence of a specialist predator: Ant invasions and horned lizards. Ecol. Appl. 12:291–298.

[ece31757-bib-0063] Suarez, A. V. , and N. D. Tsutsui . 2008 The evolutionary consequences of biological invasions. Mol. Ecol. 17:351–360. 18173507 10.1111/j.1365-294X.2007.03456.x

[ece31757-bib-0064] Takezaki, N. , M. Nei , and K. Tamura . 2010 POPTREE2: Software for Constructing Population Trees from Allele Frequency Data and Computing Other Population Statistics with Windows Interface. Mol. Biol. Evol. 27:747–752. 20022889 10.1093/molbev/msp312 PMC2877541

[ece31757-bib-0065] Thoren, P. A. , R. J. Paxton , and A. Estoup . 1995 Unusually high frequency of (CT)_n_ and (GT)_n_ microsatellite loci in a yellowjacket wasp, *Vespula rufa* (Hymenoptera: Vespidae). Insect Mol. Biol. 4:141–148. 8589840 10.1111/j.1365-2583.1995.tb00019.x

[ece31757-bib-0066] Tsuchida, K. , K. Kudo , and N. Ishiguro . 2014 Genetic structure of an introduced paper wasp, *Polistes chinensis antennalis* (Hymenoptera, Vespidae) in New Zealand. Mol. Ecol. 23:4018–4034. 25041373 10.1111/mec.12852

[ece31757-bib-0067] Tsutsui, N. D. , and A. V. Suarez . 2003 The colony structure and population biology of invasive ants. Conserv. Biol. 17:48–58.

[ece31757-bib-0068] Tsutsui, N. D. , A. V. Suarez , D. A. Holway , and T. J. Case . 2000 Reduced genetic variation and the success of an invasive species. Proc. Natl Acad. Sci. 97:5948–5953. 10811892 10.1073/pnas.100110397 PMC18539

[ece31757-bib-0069] Vargo, E. L. 2003 Hierarchical analysis of colony and population genetic structure of the eastern subterranean termite, *Reticulitermes flavipes*, using two classes of molecular markers. Evolution 57:2805–2818. 14761059 10.1111/j.0014-3820.2003.tb01522.x

[ece31757-bib-0070] Visscher, P. K. , and R. S. Vetter . 2003 Annual and multi‐year nests of the western yellowjacket, *Vespula pensylvanica*, in California. Insectes Soc. 50:160–166.

[ece31757-bib-0071] Weir, B. S. 1996 Genetic data analysis. Sinauer, Sunderland, MA.

[ece31757-bib-0072] Weir, B. S. , and C. C. Cockerham . 1984 *F*‐statistcs for the analysis of population structure. Evolution 38:1358–1370. 10.1111/j.1558-5646.1984.tb05657.x 28563791

[ece31757-bib-0073] Wilson, E. O. 1996 Hawaii: A world without social insects. Bishop Museum Occasional Papers, 3–7.

[ece31757-bib-0074] Wilson, E. E. , and D. A. Holway . 2010 Multiple mechanisms underlie displacement of solitary Hawaiian Hymenoptera by an invasive social wasp. Ecology 91:3294–3302. 21141190 10.1890/09-1187.1

[ece31757-bib-0075] Wright, S. 1943 Isolation by distance. Genetics 28:114–138. 17247074 10.1093/genetics/28.2.114 PMC1209196

